# Evaluation of a Commercial Multiplex Quantitative PCR (qPCR) Assay for Simultaneous Detection of Mycoplasma genitalium and Macrolide Resistance-Associated Mutations in Clinical Specimens

**DOI:** 10.1128/JCM.02168-16

**Published:** 2017-02-22

**Authors:** Chloé Le Roy, Nadège Hénin, Cécile Bébéar, Sabine Pereyre

**Affiliations:** a Univ. Bordeaux, USC EA 3671 Mycoplasmal and Chlamydial Infections in Humans, Bordeaux, France; b INRA, USC EA 3671 Mycoplasmal and Chlamydial Infections in Humans, Bordeaux, France; c Laboratoire de Bactériologie, Centre Hospitalier Universitaire de Bordeaux, Bordeaux, France; Mayo Clinic

**Keywords:** Mycoplasma genitalium, mycoplasma, PCR, antibiotic resistance, genitalium, macrolides, resistance, real-time PCR, urogenital specimen

## LETTER

Macrolide antibiotics are the first-line treatment for Mycoplasma genitalium infections; however, macrolide resistance has increased up to 40% in several countries (1–3). Consequently, the 2016 European guideline on M. genitalium infections has recommended complementing the molecular detection of M. genitalium with an assay capable of detecting macrolide resistance-associated mutations (4).

We aimed to evaluate the clinical performance of the CE-marked ResistancePlus MG kit (SpeeDx, Australia), which utilizes PlexZyme/PlexPrime technology (5) for the detection of M. genitalium (MgPa adhesin gene) and the five predominant 23S rRNA macrolide resistance-associated mutations (A2058G, A2059G, A2058C, A2059C, and A2058T [Escherichia coli numbering]). This was compared to in-house assays using real-time quantitative PCR (qPCR) to detect M. genitalium and real-time PCR and melting curve analysis to detect the macrolide resistance-associated mutations (“macrolide resistance qPCR”) (6).

A total of 206 male and female urogenital specimens previously analyzed using an in-house method (7) and conserved at −80°C (94 M. genitalium-positive and 112 M. genitalium-negative specimens) were retrospectively and systematically selected from samples collected in 2014 to 2015 at the Bordeaux University Hospital (France). A 5-μl volume of the internal control provided in the kit was spiked into 200 μl of specimen before extraction, which was performed using a MagNA Pure 96 instrument (Roche Diagnostics). The ResistancePlus MG and in-house assays were performed using the Cobas z480 analyzer of the Cobas 4800 platform (Roche Diagnostics), according to the instructions of the manufacturers. An additional M. genitalium detection assay, using the CE-marked S-DiaMGTV kit (Diagenode, Belgium) (8, 9), was performed such that any two of the possible three comparator results would define infection status. Pyrosequencing (1) was performed for four specimens that could not be amplified using the in-house macrolide resistance qPCR. The reference method for determining the presence of macrolide resistance-associated mutations was 23S rRNA sequencing (6). The ResistancePlus MG kit provided did not discern between absence of mutation and absence of 23S rRNA gene amplification. A 23S rRNA amplification control would be beneficial in a future version of the assay.

For M. genitalium detection, there was a 0.48% (1/206) rate of invalid results due to internal control failure. The concordance between the ResistancePlus MG kit result and the M. genitalium patient status was 99.5%, with a kappa value of 0.98. The clinical sensitivity and specificity were 98.9% and 100%, respectively (Table 1).

**TABLE 1:** Clinical performance characteristics of the ResistancePlus MG kit in comparison to M. genitalium infection status

M. genitalium infection status	ResistancePlus MG kit characteristic(s)^*a*^					
	No. of positive results	No. of negative results	% sensitivity (95% CI)	% specificity (95% CI)	PPV (95% CI)	NPV (95% CI)
Positive	94	1	98.9 (94.3–99.8)	100 (96.6–100)	100 (96.1–100)	99.1 (95.1–99.8)
Negative	0	110				

a CI, confidence interval; PPV, positive predictive value; NPV, negative predictive value.

**TABLE 2:** Clinical performance characteristics of the ResistancePlus MG kit in comparison to macrolide resistance status

Macrolide resistance status	ResistancePlus MG kit characteristic(s)^*a*^					
	No. of results with presence of mutation	No. of results with no mutation	% sensitivity (95% CI)	% specificity (95% CI)	PPV (95% CI)	NPV (95% CI)
Presence of mutation	21	1	95.4 (78.2–99.2)	95.8 (88.3–98.5)	87.5 (69.0–95.7)	98.6 (92.2–99.7)
No mutation	3	68				

a CI, confidence interval; PPV, positive predictive value; NPV, negative predictive value.

According to the M. genitalium-positive patient status, 93 M. genitalium-positive specimens were retained for the macrolide resistance evaluation (Table 2). Among them, four specimens presented discrepant results between the macrolide resistance qPCR and the ResistancePlus MG tests. Three specimens were called as mutants by the ResistancePlus MG kit but not by the macrolide resistance qPCR. Sequencing of the 23S rRNA confirmed the absence of mutations. For the remaining specimen, sequencing identified an A2062T mutation, which is not included in the ResistancePlus MG kit. Overall, the concordance between the ResistancePlus MG kit and the macrolide resistance status was 95.6%, with a kappa value of 0.88. As previously reported (5), the clinical sensitivity and specificity of the ResistancePlus MG kit were good (95.4% and 95.8%, respectively).

In conclusion, the ResistancePlus MG kit is a rapid and reliable method to simultaneously detect M. genitalium and determine macrolide resistance in clinical specimens.
